# Supplementary material for: Modeling glioblastoma heterogeneity as a dynamic network of cell states
Source: Mol Syst Biol. 2021 Sep 16;17(9):e10105. doi: 10.15252/msb.202010105 (PMC8444284; doi:10.15252/msb.202010105)
Supplement: Supplementary file 6 — Source Data for Figure 5 [file MSB-17-e10105-s004.zip › Figure5A_sourcedata/GSEA_3071/nosigdb_stateVI.GseaPreranked.1622795169056/RICHARDS_NOWAKOWSKI_SCIENCE_2017_ASTROCYTE_UPREG.html]

Details for gene set RICHARDS\_NOWAKOWSKI\_SCIENCE\_2017\_ASTROCYTE\_UPREG[GSEA]

|  || Dataset | state63071 |
| Phenotype | NoPhenotypeAvailable |
| Upregulated in class | na\_neg |
| GeneSet | RICHARDS\_NOWAKOWSKI\_SCIENCE\_2017\_ASTROCYTE\_UPREG |
| Enrichment Score (ES) | -0.53037065 |
| Normalized Enrichment Score (NES) | -2.936205 |
| Nominal p-value | 0.0 |
| FDR q-value | 0.0 |
| FWER p-Value | 0.0 |
Table: GSEA Results Summary

  

Fig 1: Enrichment plot: RICHARDS\_NOWAKOWSKI\_SCIENCE\_2017\_ASTROCYTE\_UPREG      
 Profile of the Running ES Score & Positions of GeneSet Members on the Rank Ordered List

  

| PROBE | GENE SYMBOL | GENE\_TITLE | RANK IN GENE LIST | RANK METRIC SCORE | RUNNING ES | CORE ENRICHMENT || 1 | ALCAM |  |  | 7 | 1.185 | 0.0038 | No |
| 2 | MT1E |  |  | 10 | 1.128 | 0.0089 | No |
| 3 | RGCC |  |  | 17 | 1.065 | 0.0124 | No |
| 4 | F3 |  |  | 26 | 0.988 | 0.0150 | No |
| 5 | AXL |  |  | 33 | 0.946 | 0.0179 | No |
| 6 | ADAMTS6 |  |  | 53 | 0.864 | 0.0166 | No |
| 7 | MT2A |  |  | 58 | 0.859 | 0.0197 | No |
| 8 | NOG |  |  | 77 | 0.793 | 0.0183 | No |
| 9 | LTBP1 |  |  | 84 | 0.779 | 0.0204 | No |
| 10 | HOPX |  |  | 89 | 0.761 | 0.0230 | No |
| 11 | PKM |  |  | 125 | 0.699 | 0.0161 | No |
| 12 | DLC1 |  |  | 129 | 0.696 | 0.0187 | No |
| 13 | IGFBP5 |  |  | 145 | 0.671 | 0.0176 | No |
| 14 | ENO1 |  |  | 223 | 0.587 | -0.0024 | No |
| 15 | TACC1 |  |  | 245 | 0.568 | -0.0058 | No |
| 16 | RGS20 |  |  | 259 | 0.561 | -0.0068 | No |
| 17 | RHOC |  |  | 277 | 0.551 | -0.0091 | No |
| 18 | ZFP36L2 |  |  | 284 | 0.547 | -0.0082 | No |
| 19 | JUN |  |  | 321 | 0.527 | -0.0163 | No |
| 20 | LGALS1 |  |  | 323 | 0.526 | -0.0139 | No |
| 21 | CCDC80 |  |  | 343 | 0.514 | -0.0170 | No |
| 22 | NTNG1 |  |  | 357 | 0.509 | -0.0183 | No |
| 23 | CYR61 |  |  | 377 | 0.498 | -0.0215 | No |
| 24 | TXNIP |  |  | 402 | 0.487 | -0.0262 | No |
| 25 | SSR3 |  |  | 422 | 0.480 | -0.0294 | No |
| 26 | GLUD1 |  |  | 433 | 0.476 | -0.0300 | No |
| 27 | MAST4 |  |  | 446 | 0.473 | -0.0312 | No |
| 28 | FGF1 |  |  | 562 | 0.436 | -0.0632 | No |
| 29 | DIO2 |  |  | 567 | 0.434 | -0.0623 | No |
| 30 | PHACTR2 |  |  | 580 | 0.431 | -0.0637 | No |
| 31 | EFHD2 |  |  | 598 | 0.425 | -0.0666 | No |
| 32 | DPF3 |  |  | 601 | 0.425 | -0.0651 | No |
| 33 | RCN1 |  |  | 641 | 0.413 | -0.0746 | No |
| 34 | EGFR |  |  | 669 | 0.407 | -0.0806 | No |
| 35 | PDXK |  |  | 778 | 0.381 | -0.1108 | No |
| 36 | POLR2L |  |  | 800 | 0.377 | -0.1152 | No |
| 37 | CPT1A |  |  | 832 | 0.371 | -0.1225 | No |
| 38 | MT1X |  |  | 867 | 0.366 | -0.1308 | No |
| 39 | JAG1 |  |  | 876 | 0.365 | -0.1314 | No |
| 40 | VIM |  |  | 897 | 0.361 | -0.1355 | No |
| 41 | PSME2 |  |  | 962 | 0.351 | -0.1528 | No |
| 42 | SCCPDH |  |  | 1057 | 0.336 | -0.1790 | No |
| 43 | PDLIM5 |  |  | 1062 | 0.335 | -0.1786 | No |
| 44 | TRIM47 |  |  | 1069 | 0.334 | -0.1787 | No |
| 45 | APCDD1 |  |  | 1157 | 0.323 | -0.2029 | No |
| 46 | SLC25A5 |  |  | 1163 | 0.321 | -0.2028 | No |
| 47 | GNG12 |  |  | 1178 | 0.319 | -0.2054 | No |
| 48 | TFPI |  |  | 1205 | 0.315 | -0.2115 | No |
| 49 | MEST |  |  | 1294 | 0.304 | -0.2362 | No |
| 50 | S1PR1 |  |  | 1306 | 0.302 | -0.2379 | No |
| 51 | NTM |  |  | 1320 | 0.301 | -0.2403 | No |
| 52 | EPB41L2 |  |  | 1343 | 0.299 | -0.2453 | No |
| 53 | RAB13 |  |  | 1416 | 0.290 | -0.2653 | No |
| 54 | THG1L |  |  | 1451 | 0.285 | -0.2740 | No |
| 55 | FAM120A |  |  | 1469 | 0.283 | -0.2776 | No |
| 56 | LIFR |  |  | 1567 | 0.272 | -0.3051 | No |
| 57 | TRAPPC3 |  |  | 1573 | 0.271 | -0.3052 | No |
| 58 | LRRC17 |  |  | 1593 | 0.269 | -0.3095 | No |
| 59 | SLC2A1 |  |  | 1606 | 0.268 | -0.3117 | No |
| 60 | MCFD2 |  |  | 1697 | 0.260 | -0.3372 | No |
| 61 | CHKA |  |  | 1733 | 0.257 | -0.3463 | No |
| 62 | BEND7 |  |  | 1758 | 0.255 | -0.3522 | No |
| 63 | ZIC1 |  |  | 1814 | -0.251 | -0.3673 | No |
| 64 | TAPBP |  |  | 1817 | -0.252 | -0.3666 | No |
| 65 | SDC3 |  |  | 1818 | -0.252 | -0.3654 | No |
| 66 | USP46 |  |  | 1847 | -0.256 | -0.3724 | No |
| 67 | STAT3 |  |  | 1861 | -0.259 | -0.3750 | No |
| 68 | PRNP |  |  | 1866 | -0.260 | -0.3749 | No |
| 69 | NOTCH3 |  |  | 1872 | -0.260 | -0.3750 | No |
| 70 | LRP1B |  |  | 1882 | -0.261 | -0.3764 | No |
| 71 | IFNAR1 |  |  | 1897 | -0.263 | -0.3793 | No |
| 72 | COL11A1 |  |  | 1919 | -0.267 | -0.3842 | No |
| 73 | PHLPP1 |  |  | 1937 | -0.269 | -0.3879 | No |
| 74 | SAT1 |  |  | 1952 | -0.271 | -0.3907 | No |
| 75 | PCDH17 |  |  | 1955 | -0.271 | -0.3899 | No |
| 76 | OLIG2 |  |  | 1968 | -0.274 | -0.3921 | No |
| 77 | NRBP2 |  |  | 1998 | -0.278 | -0.3994 | No |
| 78 | LRRTM3 |  |  | 2006 | -0.279 | -0.4000 | No |
| 79 | CREM |  |  | 2019 | -0.282 | -0.4022 | No |
| 80 | SNX3 |  |  | 2037 | -0.283 | -0.4058 | No |
| 81 | DOCK1 |  |  | 2063 | -0.289 | -0.4118 | No |
| 82 | SAMD8 |  |  | 2069 | -0.290 | -0.4119 | No |
| 83 | FAM84B |  |  | 2080 | -0.291 | -0.4134 | No |
| 84 | GATM |  |  | 2082 | -0.292 | -0.4122 | No |
| 85 | ALDH6A1 |  |  | 2091 | -0.294 | -0.4131 | No |
| 86 | VEGFA |  |  | 2104 | -0.298 | -0.4152 | No |
| 87 | LRP4 |  |  | 2111 | -0.300 | -0.4155 | No |
| 88 | FYN |  |  | 2118 | -0.301 | -0.4158 | No |
| 89 | PTPN13 |  |  | 2122 | -0.301 | -0.4152 | No |
| 90 | RASSF4 |  |  | 2123 | -0.301 | -0.4137 | No |
| 91 | SERPINE2 |  |  | 2128 | -0.302 | -0.4134 | No |
| 92 | PARP14 |  |  | 2145 | -0.304 | -0.4166 | No |
| 93 | FAM3C |  |  | 2162 | -0.307 | -0.4198 | No |
| 94 | TMEM132B |  |  | 2168 | -0.308 | -0.4198 | No |
| 95 | CPE |  |  | 2173 | -0.309 | -0.4194 | No |
| 96 | NPAS3 |  |  | 2184 | -0.312 | -0.4208 | No |
| 97 | NEAT1 |  |  | 2208 | -0.316 | -0.4261 | No |
| 98 | CASC4 |  |  | 2256 | -0.325 | -0.4384 | No |
| 99 | ITGA6 |  |  | 2267 | -0.327 | -0.4398 | No |
| 100 | PAX6 |  |  | 2291 | -0.331 | -0.4449 | No |
| 101 | CRYL1 |  |  | 2296 | -0.332 | -0.4445 | No |
| 102 | APC |  |  | 2301 | -0.333 | -0.4440 | No |
| 103 | ARHGAP12 |  |  | 2302 | -0.333 | -0.4423 | No |
| 104 | RHOJ |  |  | 2311 | -0.335 | -0.4430 | No |
| 105 | METTL7A |  |  | 2313 | -0.335 | -0.4417 | No |
| 106 | TNK2 |  |  | 2314 | -0.335 | -0.4400 | No |
| 107 | BMP7 |  |  | 2329 | -0.339 | -0.4425 | No |
| 108 | CTNND2 |  |  | 2331 | -0.339 | -0.4411 | No |
| 109 | STK17A |  |  | 2336 | -0.340 | -0.4406 | No |
| 110 | TMED10 |  |  | 2344 | -0.342 | -0.4409 | No |
| 111 | SEMA6D |  |  | 2377 | -0.349 | -0.4487 | No |
| 112 | ATP9B |  |  | 2378 | -0.349 | -0.4470 | No |
| 113 | SIRPA |  |  | 2398 | -0.353 | -0.4508 | No |
| 114 | ABAT |  |  | 2438 | -0.365 | -0.4606 | No |
| 115 | CSGALNACT1 |  |  | 2454 | -0.367 | -0.4632 | No |
| 116 | B2M |  |  | 2460 | -0.369 | -0.4629 | No |
| 117 | C3orf70 |  |  | 2464 | -0.370 | -0.4619 | No |
| 118 | IFI6 |  |  | 2467 | -0.370 | -0.4607 | No |
| 119 | SEPT8 |  |  | 2472 | -0.370 | -0.4600 | No |
| 120 | ITGAV |  |  | 2476 | -0.371 | -0.4591 | No |
| 121 | TMEM106B |  |  | 2479 | -0.372 | -0.4578 | No |
| 122 | MLLT6 |  |  | 2482 | -0.374 | -0.4565 | No |
| 123 | PHYHIPL |  |  | 2512 | -0.381 | -0.4632 | No |
| 124 | IDH1 |  |  | 2526 | -0.384 | -0.4652 | No |
| 125 | ADCY2 |  |  | 2537 | -0.386 | -0.4662 | No |
| 126 | SLITRK2 |  |  | 2541 | -0.387 | -0.4652 | No |
| 127 | BMPR1A |  |  | 2555 | -0.389 | -0.4671 | No |
| 128 | SPRY2 |  |  | 2569 | -0.391 | -0.4690 | No |
| 129 | ZMAT3 |  |  | 2590 | -0.399 | -0.4730 | No |
| 130 | DST |  |  | 2614 | -0.404 | -0.4778 | No |
| 131 | DSEL |  |  | 2668 | -0.415 | -0.4915 | No |
| 132 | CAMK2D |  |  | 2688 | -0.421 | -0.4950 | No |
| 133 | SEPT2 |  |  | 2703 | -0.426 | -0.4971 | No |
| 134 | PBXIP1 |  |  | 2725 | -0.431 | -0.5011 | No |
| 135 | CLDND1 |  |  | 2740 | -0.436 | -0.5031 | No |
| 136 | VEGFB |  |  | 2744 | -0.438 | -0.5018 | No |
| 137 | P2RY1 |  |  | 2772 | -0.447 | -0.5076 | No |
| 138 | RAB31 |  |  | 2806 | -0.459 | -0.5151 | No |
| 139 | BAALC |  |  | 2819 | -0.462 | -0.5164 | No |
| 140 | SPRED1 |  |  | 2825 | -0.464 | -0.5156 | No |
| 141 | TSPAN3 |  |  | 2828 | -0.465 | -0.5138 | No |
| 142 | QKI |  |  | 2844 | -0.474 | -0.5159 | No |
| 143 | TSPAN7 |  |  | 2848 | -0.475 | -0.5145 | No |
| 144 | CTNNB1 |  |  | 2863 | -0.483 | -0.5162 | No |
| 145 | HS2ST1 |  |  | 2878 | -0.486 | -0.5179 | No |
| 146 | SEMA6A |  |  | 2881 | -0.487 | -0.5161 | No |
| 147 | PON2 |  |  | 2884 | -0.490 | -0.5142 | No |
| 148 | ILDR2 |  |  | 2904 | -0.496 | -0.5174 | No |
| 149 | LAPTM4B |  |  | 2910 | -0.498 | -0.5164 | No |
| 150 | DNASE2 |  |  | 2924 | -0.503 | -0.5178 | No |
| 151 | PNISR |  |  | 2929 | -0.506 | -0.5164 | No |
| 152 | ZFYVE16 |  |  | 2933 | -0.507 | -0.5148 | No |
| 153 | PDE4B |  |  | 2948 | -0.513 | -0.5164 | No |
| 154 | GAS1 |  |  | 2953 | -0.515 | -0.5150 | No |
| 155 | TRIL |  |  | 2958 | -0.519 | -0.5136 | No |
| 156 | LGALS3BP |  |  | 2989 | -0.533 | -0.5199 | No |
| 157 | ARHGEF6 |  |  | 3003 | -0.544 | -0.5210 | No |
| 158 | PDLIM3 |  |  | 3011 | -0.546 | -0.5204 | No |
| 159 | ID2 |  |  | 3026 | -0.554 | -0.5217 | No |
| 160 | CD81 |  |  | 3056 | -0.566 | -0.5275 | Yes |
| 161 | CACNB2 |  |  | 3062 | -0.569 | -0.5262 | Yes |
| 162 | RFTN2 |  |  | 3065 | -0.570 | -0.5239 | Yes |
| 163 | PTPRA |  |  | 3073 | -0.576 | -0.5231 | Yes |
| 164 | FABP5 |  |  | 3079 | -0.577 | -0.5217 | Yes |
| 165 | RNF13 |  |  | 3089 | -0.580 | -0.5215 | Yes |
| 166 | NACC2 |  |  | 3112 | -0.594 | -0.5251 | Yes |
| 167 | PCDH9 |  |  | 3113 | -0.594 | -0.5221 | Yes |
| 168 | OLIG1 |  |  | 3126 | -0.601 | -0.5227 | Yes |
| 169 | WSB1 |  |  | 3130 | -0.608 | -0.5206 | Yes |
| 170 | MEIS1 |  |  | 3133 | -0.610 | -0.5181 | Yes |
| 171 | SCD5 |  |  | 3135 | -0.611 | -0.5153 | Yes |
| 172 | GPC6 |  |  | 3138 | -0.612 | -0.5129 | Yes |
| 173 | SPATA6 |  |  | 3143 | -0.614 | -0.5110 | Yes |
| 174 | JAM2 |  |  | 3147 | -0.615 | -0.5088 | Yes |
| 175 | ITGB8 |  |  | 3148 | -0.616 | -0.5057 | Yes |
| 176 | GOLIM4 |  |  | 3165 | -0.634 | -0.5073 | Yes |
| 177 | ST3GAL5 |  |  | 3172 | -0.639 | -0.5059 | Yes |
| 178 | FADS2 |  |  | 3173 | -0.640 | -0.5027 | Yes |
| 179 | OLFM2 |  |  | 3176 | -0.641 | -0.5001 | Yes |
| 180 | SRI |  |  | 3179 | -0.643 | -0.4975 | Yes |
| 181 | TIMP2 |  |  | 3180 | -0.643 | -0.4943 | Yes |
| 182 | ZMYM2 |  |  | 3181 | -0.643 | -0.4911 | Yes |
| 183 | ITM2C |  |  | 3187 | -0.652 | -0.4893 | Yes |
| 184 | AASS |  |  | 3191 | -0.657 | -0.4869 | Yes |
| 185 | NOTCH1 |  |  | 3192 | -0.657 | -0.4837 | Yes |
| 186 | STON2 |  |  | 3220 | -0.688 | -0.4882 | Yes |
| 187 | TRPS1 |  |  | 3232 | -0.698 | -0.4880 | Yes |
| 188 | AIF1L |  |  | 3235 | -0.704 | -0.4851 | Yes |
| 189 | PSAP |  |  | 3252 | -0.714 | -0.4863 | Yes |
| 190 | CERS6 |  |  | 3258 | -0.718 | -0.4842 | Yes |
| 191 | MAGI1 |  |  | 3265 | -0.726 | -0.4824 | Yes |
| 192 | SOX9 |  |  | 3280 | -0.739 | -0.4828 | Yes |
| 193 | CXXC5 |  |  | 3287 | -0.745 | -0.4809 | Yes |
| 194 | GNG7 |  |  | 3310 | -0.774 | -0.4836 | Yes |
| 195 | SESN3 |  |  | 3311 | -0.775 | -0.4797 | Yes |
| 196 | SOX2 |  |  | 3314 | -0.778 | -0.4764 | Yes |
| 197 | SALL3 |  |  | 3318 | -0.784 | -0.4734 | Yes |
| 198 | MLC1 |  |  | 3326 | -0.793 | -0.4715 | Yes |
| 199 | NCAN |  |  | 3329 | -0.794 | -0.4681 | Yes |
| 200 | HTRA1 |  |  | 3331 | -0.796 | -0.4644 | Yes |
| 201 | DAG1 |  |  | 3336 | -0.803 | -0.4616 | Yes |
| 202 | TGFB2 |  |  | 3340 | -0.804 | -0.4585 | Yes |
| 203 | DBI |  |  | 3349 | -0.812 | -0.4568 | Yes |
| 204 | DDR1 |  |  | 3350 | -0.814 | -0.4528 | Yes |
| 205 | ZEB1 |  |  | 3363 | -0.827 | -0.4522 | Yes |
| 206 | NLGN1 |  |  | 3364 | -0.831 | -0.4480 | Yes |
| 207 | SRGAP2B |  |  | 3372 | -0.841 | -0.4459 | Yes |
| 208 | ETV1 |  |  | 3378 | -0.857 | -0.4431 | Yes |
| 209 | WSCD1 |  |  | 3379 | -0.860 | -0.4388 | Yes |
| 210 | TGIF1 |  |  | 3380 | -0.860 | -0.4345 | Yes |
| 211 | RHOBTB3 |  |  | 3400 | -0.893 | -0.4357 | Yes |
| 212 | BCHE |  |  | 3405 | -0.909 | -0.4324 | Yes |
| 213 | NLGN4X |  |  | 3412 | -0.918 | -0.4296 | Yes |
| 214 | PRCP |  |  | 3417 | -0.927 | -0.4261 | Yes |
| 215 | SOX8 |  |  | 3418 | -0.934 | -0.4215 | Yes |
| 216 | LRRN1 |  |  | 3420 | -0.942 | -0.4170 | Yes |
| 217 | LDLRAD3 |  |  | 3424 | -0.946 | -0.4132 | Yes |
| 218 | FJX1 |  |  | 3437 | -0.976 | -0.4119 | Yes |
| 219 | MAML2 |  |  | 3441 | -0.983 | -0.4079 | Yes |
| 220 | TRIM9 |  |  | 3442 | -0.985 | -0.4030 | Yes |
| 221 | CD9 |  |  | 3445 | -0.995 | -0.3986 | Yes |
| 222 | SLC1A3 |  |  | 3458 | -1.025 | -0.3970 | Yes |
| 223 | GPRC5B |  |  | 3459 | -1.026 | -0.3919 | Yes |
| 224 | PTPRG |  |  | 3461 | -1.034 | -0.3870 | Yes |
| 225 | ETV5 |  |  | 3466 | -1.043 | -0.3830 | Yes |
| 226 | CHPT1 |  |  | 3470 | -1.048 | -0.3787 | Yes |
| 227 | PCDHGC3 |  |  | 3472 | -1.051 | -0.3737 | Yes |
| 228 | FOS |  |  | 3476 | -1.056 | -0.3693 | Yes |
| 229 | PLEKHH2 |  |  | 3478 | -1.060 | -0.3643 | Yes |
| 230 | TTYH1 |  |  | 3481 | -1.067 | -0.3596 | Yes |
| 231 | TRIB2 |  |  | 3488 | -1.088 | -0.3560 | Yes |
| 232 | LITAF |  |  | 3491 | -1.089 | -0.3511 | Yes |
| 233 | FAT1 |  |  | 3496 | -1.107 | -0.3468 | Yes |
| 234 | RNF180 |  |  | 3497 | -1.113 | -0.3412 | Yes |
| 235 | FGFBP3 |  |  | 3502 | -1.120 | -0.3368 | Yes |
| 236 | NOVA1 |  |  | 3510 | -1.148 | -0.3331 | Yes |
| 237 | MMP16 |  |  | 3511 | -1.150 | -0.3274 | Yes |
| 238 | DUSP6 |  |  | 3512 | -1.151 | -0.3217 | Yes |
| 239 | GPM6A |  |  | 3515 | -1.169 | -0.3164 | Yes |
| 240 | MYO10 |  |  | 3516 | -1.170 | -0.3106 | Yes |
| 241 | SPECC1 |  |  | 3523 | -1.202 | -0.3063 | Yes |
| 242 | DCLK2 |  |  | 3524 | -1.206 | -0.3003 | Yes |
| 243 | SEMA5A |  |  | 3526 | -1.210 | -0.2946 | Yes |
| 244 | RASSF2 |  |  | 3527 | -1.212 | -0.2885 | Yes |
| 245 | NPNT |  |  | 3533 | -1.229 | -0.2839 | Yes |
| 246 | LSAMP |  |  | 3537 | -1.257 | -0.2785 | Yes |
| 247 | ITM2B |  |  | 3540 | -1.269 | -0.2727 | Yes |
| 248 | SEL1L3 |  |  | 3549 | -1.336 | -0.2684 | Yes |
| 249 | ITPR2 |  |  | 3556 | -1.388 | -0.2633 | Yes |
| 250 | CDCA7L |  |  | 3557 | -1.393 | -0.2563 | Yes |
| 251 | LIMA1 |  |  | 3564 | -1.432 | -0.2510 | Yes |
| 252 | ZBTB20 |  |  | 3567 | -1.435 | -0.2444 | Yes |
| 253 | SLC35F1 |  |  | 3576 | -1.554 | -0.2390 | Yes |
| 254 | CADM4 |  |  | 3577 | -1.560 | -0.2312 | Yes |
| 255 | CLSTN1 |  |  | 3578 | -1.562 | -0.2234 | Yes |
| 256 | FAM181B |  |  | 3582 | -1.594 | -0.2163 | Yes |
| 257 | MAP3K1 |  |  | 3584 | -1.605 | -0.2086 | Yes |
| 258 | TNC |  |  | 3586 | -1.618 | -0.2008 | Yes |
| 259 | SYT11 |  |  | 3587 | -1.620 | -0.1927 | Yes |
| 260 | SLC1A2 |  |  | 3589 | -1.634 | -0.1849 | Yes |
| 261 | CLU |  |  | 3601 | -1.801 | -0.1792 | Yes |
| 262 | CXCL14 |  |  | 3605 | -1.867 | -0.1707 | Yes |
| 263 | LRRN3 |  |  | 3606 | -1.871 | -0.1614 | Yes |
| 264 | SCRG1 |  |  | 3613 | -2.165 | -0.1523 | Yes |
| 265 | GPM6B |  |  | 3616 | -2.257 | -0.1417 | Yes |
| 266 | GFAP |  |  | 3619 | -2.337 | -0.1306 | Yes |
| 267 | ID4 |  |  | 3620 | -2.343 | -0.1189 | Yes |
| 268 | PMP2 |  |  | 3622 | -2.497 | -0.1067 | Yes |
| 269 | S100B |  |  | 3624 | -2.502 | -0.0945 | Yes |
| 270 | C1orf61 |  |  | 3626 | -2.616 | -0.0817 | Yes |
| 271 | FABP7 |  |  | 3629 | -2.788 | -0.0684 | Yes |
| 272 | PTN |  |  | 3632 | -2.826 | -0.0549 | Yes |
| 273 | IGFBP2 |  |  | 3634 | -3.295 | -0.0387 | Yes |
| 274 | PDGFRA |  |  | 3636 | -3.635 | -0.0209 | Yes |
| 275 | PTPRZ1 |  |  | 3637 | -4.237 | 0.0003 | Yes |
Table: GSEA details [plain text format]

  

Fig 2: RICHARDS\_NOWAKOWSKI\_SCIENCE\_2017\_ASTROCYTE\_UPREG: Random ES distribution      
 Gene set null distribution of ES for **RICHARDS\_NOWAKOWSKI\_SCIENCE\_2017\_ASTROCYTE\_UPREG**
